# Generation of Transcript Length Variants and Reprogramming of mRNA Splicing During Atherosclerosis Progression in ApoE-Deficient Mice

**DOI:** 10.3390/biomedicines12122703

**Published:** 2024-11-26

**Authors:** Miguel Hueso, Adrián Mallén, Estanis Navarro

**Affiliations:** 1 Experimental Nephrology Lab, Institut d’Investigació Biomèdica de Bellvitge-IDIBELL, C/Feixa Llarga s/n, L’Hospitalet de Llobregat, 08907 Barcelona, Spain; amallen@idibell.cat; 2 Department of Nephrology, Hospital Universitari and Bellvitge, Institut d’Investigació Biomèdica de Bellvitge-IDIBELL, C/Feixa Llarga s/n, L’Hospitalet de Llobregat, 08907 Barcelona, Spain; 3 REMAR Group, Germans Trias i Pujol Research Institute (IGTP), Ctra de Can Ruti, Camí de les Escoles s/n, 08916 Badalona, Spain

**Keywords:** 5′UTR/3′UTR lengthening, alternative splicing signatures, atherosclerosis, mRNA isoform switching, splicing reprogramming

## Abstract

**Background.** Variant 3′UTRs provide mRNAs with different binding sites for miRNAs or RNA-binding proteins (RBPs) allowing the establishment of new regulatory environments. Regulation of 3′UTR length impacts on the control of gene expression by regulating accessibility of miRNAs or RBPs to homologous sequences in mRNAs. **Objective.** Studying the dynamics of mRNA length variations in atherosclerosis (ATS) progression and reversion in ApoE-deficient mice exposed to a high-fat diet and treated with an αCD40-specific siRNA or with a sequence-scrambled siRNA as control. **Methods.** We gathered microarray mRNA expression data from the aortas of mice after 2 or 16 weeks of treatments, and used these data in a Bioinformatics analysis. **Results.** Here, we report the lengthening of the 5′UTR/3′UTRs and the shortening of the CDS in downregulated mRNAs during ATS progression. Furthermore, treatment with the αCD40-specific siRNA resulted in the partial reversion of the 3′UTR lengthening. Exon analysis showed that these length variations were actually due to changes in the number of exons embedded in mRNAs, and the further examination of transcripts co-expressed at weeks 2 and 16 in mice treated with the control siRNA revealed a process of mRNA isoform switching in which transcript variants differed in the patterns of alternative splicing or activated latent/cryptic splice sites. **Conclusion.** We document length variations in the 5′UTR/3′UTR and CDS of mRNAs downregulated during atherosclerosis progression and suggest a role for mRNA splicing reprogramming and transcript isoform switching in the generation of disease-related mRNA sequence diversity and variability.

## 1. Background

The cell nucleus is pervasively transcribed into a plethora of different RNA molecules with different coding, structural or regulatory roles [1], among them microRNAs (miRNAs). Mature miRNAs are small RNAs (over 20 nucleotides long) that exert a post-transcriptional control of gene expression by promoting mRNA degradation or by repressing translation through their binding to homologous sequences mainly at the 3′ Untranslated Regions (3′UTRs) of mRNAs [2]. 3′UTRs not only protect mRNAs from exonucleolytic digestion but also provide functional platforms for the miRNA regulation of mRNA function [3,4], which furthermore adapt to new regulatory requirements by changing the lengths of their sequences and the arrangement of regulatory motifs they harbor. In this way, sequence changes in 3′UTRs result in a deregulation of gene expression, linked to the loss (or acquisition) of specific miRNA-binding sites, which eventually could lead to diseases [5].

3′UTRs are highly polymorphic in length and sequence, and variant 3′UTRs provide mRNAs with different binding sites for miRNAs or RNA-binding proteins (RBPs), which would contribute to the establishment of new regulatory environments [6]. The regulation of 3′UTR length is thus critical for the control of gene expression by its potential to regulate the accessibility of miRNAs or RBPs to their cognate sequences in mRNAs. In this way, mRNA isoforms with alternative 3′UTRs have been seen to have differential mRNA stability [7], microRNA-binding potential [8] or interactions with competing endogenous RNAs (ceRNAs) [9]. Alternative polyadenylation is one important mechanism for regulating 3′UTR length [10]. Pre-messenger RNAs (pre-mRNAs) harbor specific polyadenylation signals (AAUAAA in its canonical form) over 30 nts upstream the end of their 3′UTRs to precisely position the cleavage/polyadenylation complex (CPSF) next the cleavage site where the Poly(A)-Binding Protein Nuclear 1 (PABPN) will extend the poly-A tail [11]. Over 70% of human genes have more than one polyadenylation site in their 3′UTRs, and 50% have three or more [12], thus making the generation of alternative 3′UTRs by alternative polyadenylation (APA) a widespread mechanism for the generation of transcript variants that are heterogeneous in length and have different regulatory potentials [13,14]. On the other hand, alternative splicing has been shown to be also involved in the regulation of 3′UTR lengths and their regulatory potentials, through different mechanisms such as intron retention, exon skipping, the incorporation of one of two mutually-exclusive terminal exons of different lengths, the use of 5′/3′ alternative sites or the activation of cryptic splice sites [3,15].

The emerging picture on alternative 3′UTRs highlights a complex collection of isoforms whose individual expression switches in a controlled way during cell proliferation and differentiation but that could be de-regulated under stress conditions. This would impact the RNA interactome, causing a mis-control of mRNA function, and facilitating the loss of physiologic homeostasis. In this way, 3′UTR shortening would increase mRNA stability by relaxing the protein or miRNA-based mechanisms of mRNA degradation, while 3′UTR lengthening would strengthen accessibility to miRNAs [16].

On the other hand, 5′UTRs are also heterogeneous in length and play a key role in regulating translation efficiency and mRNA stability [17] since they contain a number of regulatory elements and miRNA-binding sites [18], Piwi-associated RNA (piRNA)-binding sites [19], translation-regulating secondary structures [20] and protein-binding sites [21], whose alterations could eventually lead to disease [18].

Our group is interested in studying the disease-dependent alterations in the regulatory circuitry controlling mRNA stability and function, more specifically in the interactions among miRNAs/sponging ceRNAs and mRNAs. In a previous work, we described the association of adverse cardiovascular events in hemodialysis patients to SNPs in the gene of the lncRNA ANRIL [22]. Furthermore, we took advantage of the atherosclerosis-prone ApoE^-/-^ mouse model to investigate the role of CD40 signaling on atherosclerosis (ATS) progression. While in untreated mice ATS progression was evidenced by an increase in the number and extension of plaque areas in “en face” whole aortas stained with Oil-Red-O, as well as by an enlargement in the size of lesion areas in aortic sinus and ascendent aorta [23], mice treated with the αCD40 siRNA for 16 weeks showed a significantly reduced extension and severity of the atherosclerotic lesions. Furthermore, treatment also resulted in a diminished infiltration of macrophages (F4/80^+^ cells; galectin-3^+^ cells) in the intima of atherosclerotic plaques and in the downregulation of miR-125b, Taf3, Xpr1 and Ikkβ, which were significantly upregulated during ATS progression [23].

Interestingly, while studying possible targets of miR-125, we found that accession to its binding sites in the mRNAs of *Cd34* or of the scavenger receptor *SCARB1* was regulated by alternative splicing [24,25]. In the case of *Cd34*, the inclusion of an alternative exon immediately upstream of the miR-125-binding sequence activated an upstream stop-codon that led to a long 3′UTR that included the miR-125-binding sequence, while the usage of a downstream internal/cryptic splice site led to the activation of a downstream stop codon, to a short 3′UTR and to the displacement of the miR-125-binding site to the end of the coding sequence [24]. On the other side, the *SCARB1* gene includes two mutually exclusive 3′UTRs, of different lengths, of which only one has a miR-125-binding site [25]. These results prompted us to perform a global study of length variation (including 5′UTR, CDS and 3′UTR domains) in transcripts downregulated during ATS progression and regression in the above ApoE^-/-^ model of ATS, and here we present the first analysis of the results obtained. These highlight mRNA isoforms switching and the de-regulation of alternative splicing as mechanisms involved in the generation of length variability in the 5′/3′UTRs or CDS of transcripts in ATS progression. The consequences of this length variability on the regulatory circuitry controlling mRNA stability and function are discussed.

## 2. Materials and Methods

### 2.1. Mice, Tissue and RNA Extraction

**Figure 1:**
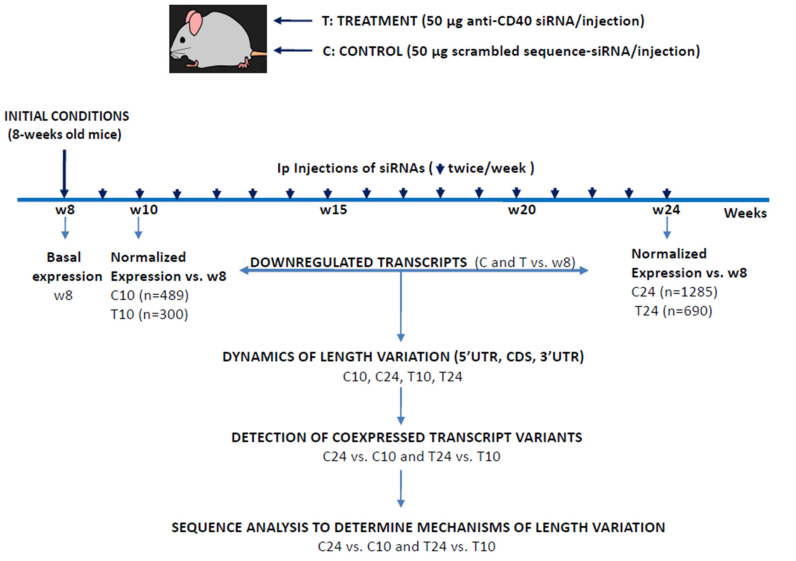
Pipeline of the experimental approach followed. Female ApoE^-/-^ mice were treated twice weekly for 16 weeks (arrowheads) with an intraperitoneal administration of 50 μg of an αCD40 siRNA (treatment group, T) or 50 μg of a scrambled sequence siRNA (control group, C). Aortic tissue was extracted at weeks 8 (basal) and 10 and 24 for both experimental groups (C10, C24, T10 and T24). Total RNA was extracted and used for a microarray experiment in which expression data were normalized to the basal levels at week 8. Only downregulated transcripts of the C and T groups were used in this analysis of length dynamics (see text). In parenthesis is the number of transcripts from each group. Transcripts simultaneously expressed in two experimental conditions (C10/C24 and T10/T24) were identified and used for bioinformatic analysis of the mechanisms regulating transcript length variation.

In this work, we used 30 female ApoE^-/-^ mice (homozygous Apoe^tm1Unc^ in the C57BL/6J background, (Jackson Laboratory, Charles River, Wilmington, MA, USA). Mice were treated twice weekly for 16 weeks with an intraperitoneal administration of 50 μg of an αCD40 siRNA (group T, for treatment, *n* = 15) or 50 μg of a scrambled siRNA as control (group C, for control, *n* = 10) and were euthanized [23]. Immediately after death, aortic tissue was extracted from mice of the basal group at week 8 (*n* = 5), for the control group (ATS progression) at weeks 10 (C10, *n* = 5) and 24 (C24, *n* = 10) and for the treatment group (αCD40 siRNA) also at weeks 10 (T10, *n* = 5) and 24 (T24, *n* = 5) (see Figure 1). Aortic tissue was then split into two pieces, one of which was used for total RNA extraction as described [23]. All animal studies were in accordance with EU guidelines on animal care, and the protocols here used were approved by the ethics committee for animal research of the University of Barcelona-HUB [CEEA, Comité Ètic d’Experimentació Animal], under the procedure name “Immunosuppressió de l’expressió de CD40 mitjançant un siRNA en un model d’arteriosclerosis en ratolins ApoE KO i evaluació dels efectes sobre la malaltía i la seva evolució”, with the approval code: Bell 457/13 and the approval date of 27/06/2013. Details on this experiment, including the pathological progression of control mice and the effects of the αCD40 siRNA therapy, have been already published [23].

### 2.2. Microarray Expression Analysis

Total RNA extracted from aortas at the different time points (10 and 24 weeks, groups C and T) were used for microarray expression analysis. In this work, we used the “Arraystar Mouse LncRNA Microarray v2.0” from Arraystar Inc. (Rockville, MD, USA) on a custom basis. This microarray was designed for the global profiling of mouse-protein-coding transcripts and LncRNAs and included 25,376 coding transcripts, where each transcript was represented by a specific exon or splice junction probe that can identify individual transcripts accurately. The output of the microarray experiment included differentially expressed mRNAs with statistical significance that passed Volcano Plot filtering with a *p*-value <= 0.05 and a Fold Change >= 2.0. Fold Changes were expressed in absolute values and all expression data were normalized to initial levels at week-8 [23].

### 2.3. Sequence Identifiers, Definitions and Nomenclature

In this analysis, we used the “seqname” and “GeneSymbol” of the different mRNA species analyzed. The “seqname” is analogous to the “Refseq” of the NCBI and was used as a unique identifier for each individual mRNA-transcribed isoform, while the “GeneSymbol” identified a gene with all its different variants. Expression was measured by the “FoldChange [FC(Abs)]” or absolute ratio (no log scale) of normalized intensities between two conditions (experimental vs. initial w8), while its statistical significance (as PValue) was calculated from *t*-test.

### 2.4. Data Analysis

We first identified downregulated transcripts that appeared in the two time points (weeks 10 and 24) for each one of the two experimental conditions (groups C and T) by using the sorting function of Excel spreadsheets. Briefly, the two lists of downregulated transcripts (weeks 10 and 24) were sequentially loaded onto the same Excel column and alphabetically sorted according to their “GeneSymbol”. The mixed list was examined looking for transcript isoforms that shared the same “GeneSymbol” but had different “seqnames”.

### 2.5. Structural Analysis

Length analysis of total transcripts, their CDS, 5′UTR and 3′UTRs, was performed at the “ShinyGO v0.76.2” browser [26] at https://bioinformatics.sdstate.edu/go/ (accessed on 19 September 2024). This program used Chi-squared and Student’s *t*-tests to identify significant length differences among a list of query transcripts and the list of exons from all protein-coding genes in the murine genome. Distribution of transcript/subdomain lengths was measured as a “density” function to represent the distribution of quantitative data against a continuous variable, in this case, sequence length in bp.

## 3. Results

We are interested in the generation of 3′UTR variants in coding transcripts as a mechanism to modulate the accession of miRNAs to their target sequences. For this work, we used the expression data previously generated on ATS progression/regression in the siCD40/ApoE^-/-^ model of mice exposed to a high-fat diet [23]. In this disease model, 8-week-old ApoE-deficient mice were treated with a scrambled-sequence siRNA as control (group control, C) or with an αCD40 siRNA as anti-ATS treatment (group treatment, T), and RNA samples were extracted from aortas at weeks 10 or 24 of age (Figure 1). Upon the microarray analysis of expression, we detected 489 transcripts downregulated in the C10 group, 1285 in the C24 group, 300 in the T10 group and the other 690 in the T24 group. Here, we will only describe the results obtained with the downregulated transcripts because the group of upregulated mRNAs from the same experiment includes a substantially much higher number of transcripts, which makes their analysis more cumbersome.

In a parallel work, we identified miR-125b as a mediator highly upregulated in ATS progression and whose expression was normalized upon αCD40 siRNA treatment [23]. Since the first analysis revealed length variations in the 3′UTRs of miR-125b-predicted targets, we underwent a more systematic study of the ATS-3′UTRome. Transcripts downregulated at weeks 10 and 24 in the control and treatment groups were submitted to a structural study that was followed by the analysis of the dynamics of length variation in ATS progression (C24 vs. C10) and treatment-related ATS regression (T24 vs. T10/T24 vs. C24). Figure 1 draws the pipeline of the experimental approach.

### 3.1. 3′UTR Lengthening in ATS Progression Is Partially Reversed by the αCD40 siRNA Treatment

**Figure 2:**
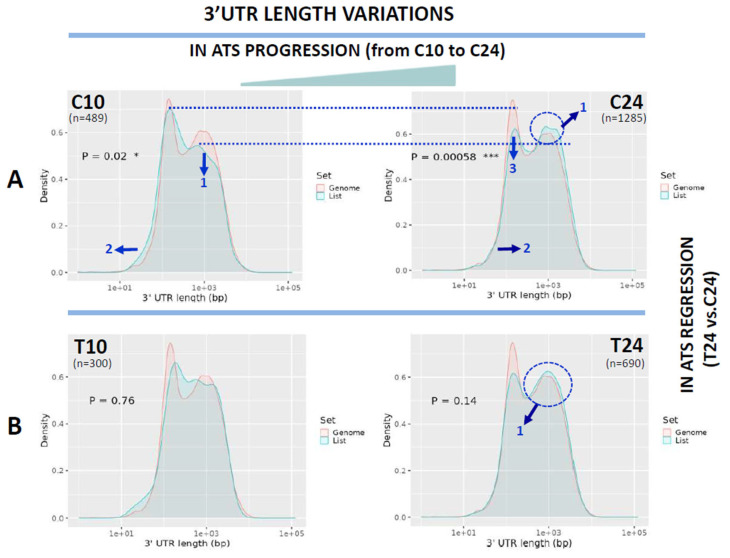
Analysis of 3′UTR length variations. Shown are the length distribution of transcripts downregulated in ATS progression (C10 to C24, panels **A**) and treatment-induced regression (T10 vs. T24, panels **B**). In all cases, the plots show the distribution of the 3′UTRs of the transcripts as transcript density vs. length in bp. The pink line refers to the distribution of the mouse reference exome, while the blue line shows that of the query population of transcripts. Blue arrows show the displacements of the blue line from the mouse reference transcriptome distribution. Dotted blue lines are drawn to facilitate comparison of peaks. Blue circles highlight differences in the second peak. Numbers inside the graphics show the statistical significance of the comparison (from Student’s *t*-test in the ShinyGO webtool; statistical significance as follows, * *p* < 0.05; *** *p* < 0.0005). In parenthesis, the number of transcripts used for the analysis in each group is noted.

We studied length variations of the 5′UTR, CDS and 3′UTRs on the ShinyGO server, which compares transcript lengths from a query list (“List”) with those from the reference murine exome from protein-coding genes. As shown in Figure 2, results are represented as superposed plots of “density vs. length in bp” in which the query is plotted in blue while the control is drawn in red, with *t*-tests identifying pairs of plots (list vs. control) that are statistically different among them. In our analysis, we used as query the lists of downregulated mRNAs at 10 and 24 weeks from the control (C, ATS progression) and from the treatment (T, ATS regression) groups of the siCD40/ApoE^-/-^ experiment. Furthermore, we used the same data to characterize changes in the patterns of mRNA isoform expression by taking advantage of the double identification (GeneSymbol/seqname) of transcripts in the microarray outputs in which the “seqname” was used as a unique identifier of each individual mRNA isoform while the “GeneSymbol” was the common identifier to all mRNA isoforms transcribed from a single gene. This allowed the identification of expression switches in which one mRNA isoform was replaced by another one from the same gene and facilitated the study of the mechanisms involved.

We first compared total mRNA lengths of the reference murine exome with those of the downregulated transcripts identified at C10 (489 transcripts), C24 (1285 transcripts), T10 (300 transcripts) and T24 (690 transcripts), but no significant length differences were found among the reference and C24, T10 or T24, and only residual differences were found for C10 (*p* = 0.033). We next studied the dynamics of the 3′UTRs in ATS progression, as well as the effects of the αCD40 siRNA treatment on this. As can be seen in Figure 2, the length distribution of 3′UTRs in the global murine exome followed a bimodal distribution with two peaks at approx. 100 bp and 1000 bp. ATS progression (C10 to C24) was characterized by a 3′UTR lengthening, from a “short” distribution in C10 with a reduced density of longer 3′UTRs in the second peak (Panel C10, Figure 2A, arrow #1) and a general shift of the query curve to the left (Panel C10, Figure 2A, arrow #2), to a “long” distribution in C24 characterized by a clear reduction in the density of 3′UTRs around 100 bp (Panel C24, Figure 2A, arrow #3), an increase in the density of 3′UTRs around 1000 bp (Panel C24, Figure 2A, arrow #1) and a general shift to the right of the query curve (Panel C24, Figure 2A, arrow #2).

On the other hand, the αCD40 siRNA treatment partially reversed the above changes detected in 3′UTR length distribution, with Figure 2B showing that 3′UTR length distribution in T10 and T24 almost recovered the normal shape of the control murine exome (*p* = n.s. among the query and the murine exome).

### 3.2. Length Changes in 5′UTRs and Coding Regions (CDS)

**Figure 3:**
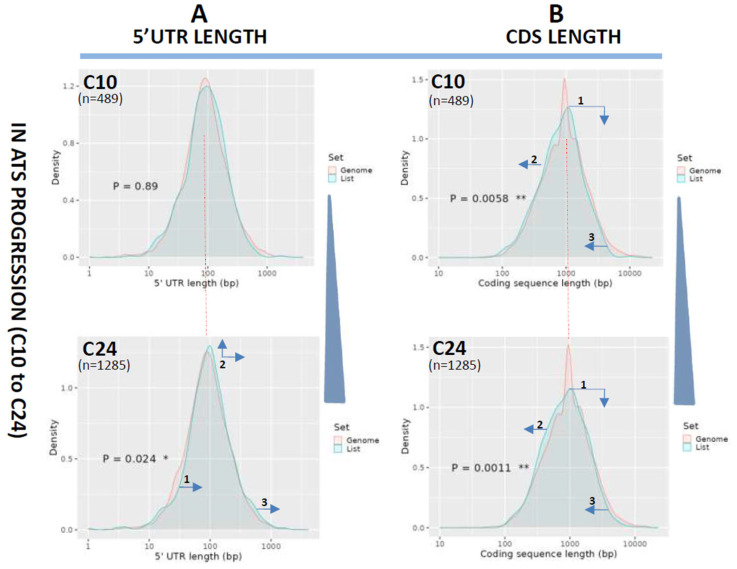
Analysis of 5′UTR and CDS length variations in ATS progression (C10 to C24). Length distribution of the 5′UTRs (**A**) or CDS (**B**) of the transcripts downregulated in ATS progression (C10 to C24) and plotted as transcript density vs. length in bp. The pink line plots the length distribution of the mouse reference exome, while the blue line shows that of the query population of transcripts. Blue arrows show the displacements of the blue line from the mouse reference exome distribution. Dotted red line centers the peak of the mouse reference exomes to facilitate comparison among plots. Numbers inside the graphics show the statistical significance of the comparison (from Student’s *t*-test in the ShinyGO webtool; statistical significance as follows, * *p* < 0.05; ** *p* < 0.005). In parenthesis, the number of transcripts used for the analysis in each group is noted.

Next, we compared 5′UTR lengths in ATS progression (C24 vs. C10 and reference). The length distribution of 5′UTRs in the reference exome followed a normal curve peaking at 100 bp (red plots at Figure 3A), essentially the same distribution of C10 (*p* = ns, Figure 3A, blue plot, upper panel). On the contrary, query transcripts at C24 showed a slight but significant shift to longer 5′UTRs as seen by the displacement of sections of the peak to the right (arrows in Figure 3A, bottom panel, blue plot). When lengths of the coding sequences were analyzed in ATS progression, a clear, significant, CDS shortening was detected for C10 and especially C24 when compared with the reference exome as seen by the shift of the query plots to the left (Figure 3B, arrows).

**Figure 4:**
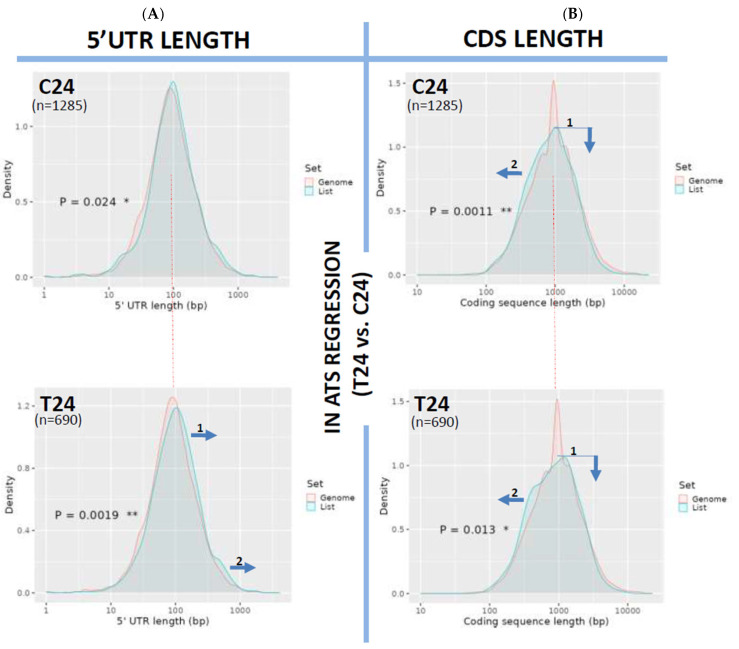
Analysis of 5′UTR and CDS length variations in ATS regression (T24 vs. C24). Length distribution of the 5′UTRs (**A**) or CDS (**B**) of the transcripts downregulated in ATS regression (T24 vs. C24) and plotted as transcript density vs. length in bp. The pink line plots the length distribution of the mouse reference exome, while the blue line shows that of the query population of transcripts. Blue arrows show the displacements of the blue line from the mouse reference exome distribution. Dotted red line centers the peak of the mouse reference exomes to facilitate comparison among plots. Numbers inside the graphics show the statistical significance of the comparison (from Student’s *t*-test in the ShinyGO webtool; statistical significance as follows, * *p* < 0.05; ** *p* < 0.005). In parenthesis, the number of transcripts used for the analysis in each group is noted.

Lastly, the lengths of 5′UTR and CDS were also examined in the treatment-induced ATS regression samples (T24 vs. C24 and reference). 5′UTRs at T24 were found to be significantly displaced towards longer sequences (*p* = 0.0019, Figure 4A, arrows in bottom panel), suggesting that the siRNA treatment did not have any effect on the normalization of the length of 5′UTRs. A similar result was obtained for the CDS length in T24 (*p* = 0.013, Figure 4B, bottom panel), also evidencing a lack of an effect of the treatment on the normalization of CDS length.

### 3.3. Variations in the Number of Exons per Coding Gene During ATS Progression and Treatment-Induced Regression

**Figure 5:**
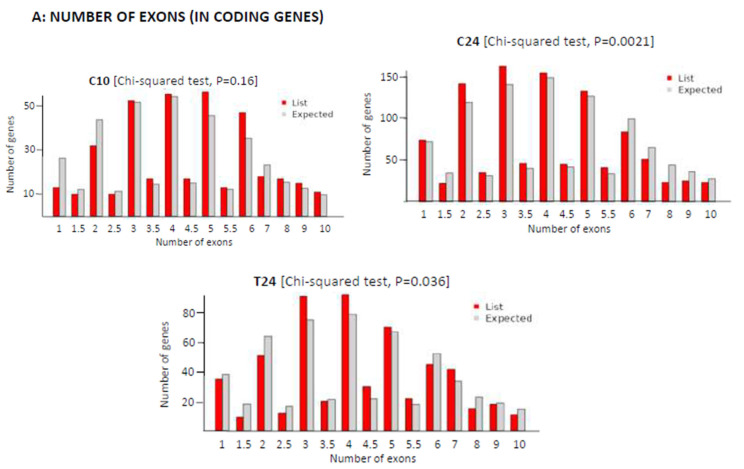
Variations in the number of exons in the groups tested compared with expected values from mouse reference transcriptome. Red bars correspond to the query group while grey bars indicate the expected values. Decimal values refer to the average number of exons across transcript variants of a gene with multiple alternatively spliced isoforms (Chi-squared test in ShinyGO).

We next aimed to study the mechanisms behind length variation during ATS progression and regression. Firstly, we analyzed exon density in the transcripts downregulated at C10, C24 and T24. Figure 5 displays the plots showing the actual number of expressed exons in coding genes (up to 10 exons, red bars), compared with the expected values from the mouse transcriptome (grey bars) and document variations in the number of exons per coding gene from a distribution similar to that of the control in C10 (*p* = 0.16) to a significant difference (*p* = 0.0021) in C24, when compared with the control, and with an increase in the number of genes with few exons (1, 2 or 3 exons) and a diminution in the number of genes with more exons (8, 9 or 10 exons) when compared with C10. The treatment with the αCD40 siRNA partially reversed this effect (T24, *p* = 0.036). Taken together, these results indicated the presence of changes in the exonic composition of transcripts during ATS progression and treatment-induced regression, pointing out to pre-mRNA splicing as the likely mechanism generating transcript variability.

### 3.4. Differential Expression of Transcript Isoforms During ATS Progression or Treatment-Induced Regression

The hypothetical impact of alternative splicing on the generation of mRNA variants was tested by studying isoform expression swifts from C10 to C24, or from T10 to T24. For ATS progression, we loaded onto an Excel spreadsheet the full list of transcripts expressed in C10 (489 entries) and C24 (1285 entries), then we mixed both lists and sorted the resulting list (1774 transcripts) by their “GeneSymbols”. Upon careful examination, looking for “GeneSymbol” entries common to the two lists, we were able to detect 114 genes simultaneously downregulated in C10 and C24. Although most of these corresponded to a single mRNA isoform (Seqname) expressed in both conditions, we detected seven cases of isoform switching. In six of them, a new transcript isoform was expressed “de novo” in C24 (Dusp22, Mta3, Rab11fip5, Rbmx, Skor and Srsf5), while, in Ly6e, two of the three isoforms expressed in C10 were silenced in C24 so that only one isoform was expressed in C24. Table 1 summarizes the length variations of the new transcript variants and their functional regions at C24, compared with those expressed at C10. Variations are encoded as longer (L), shorter (S) or equal length (=), and, as can be seen, the isoforms differentially expressed in C24 vs. C10 showed length variability mostly in their 5′UTR and CDS, with cryptic or alternative splicing accounting for most of the variability found. Supplementary Table S1 shows the real length values of all these regions, and their expression levels, and identifies transcript variants.

The same analysis was performed for T10 (300 entries) and T24 (690 entries) and yielded 153 genes simultaneously downregulated in the two conditions, most of them corresponding to isoforms expressed in the two conditions, although we also detected three cases in which a new isoform was expressed “de novo” in T24 (Ilf3, Pwwp2a and Rbmx, Table 2). Length analysis for these three new isoforms also highlighted variability in their 5′UTR, CDS and 3′UTR, with alternative or cryptic splicing being the most frequent mechanism of action. Supplementary Table S2 shows the real length values of all these regions, and their expression levels, and identifies transcript variants.

### 3.5. Dysregulation of 3′UTR Splicing Variants in ATS Progression

**Figure 6:**
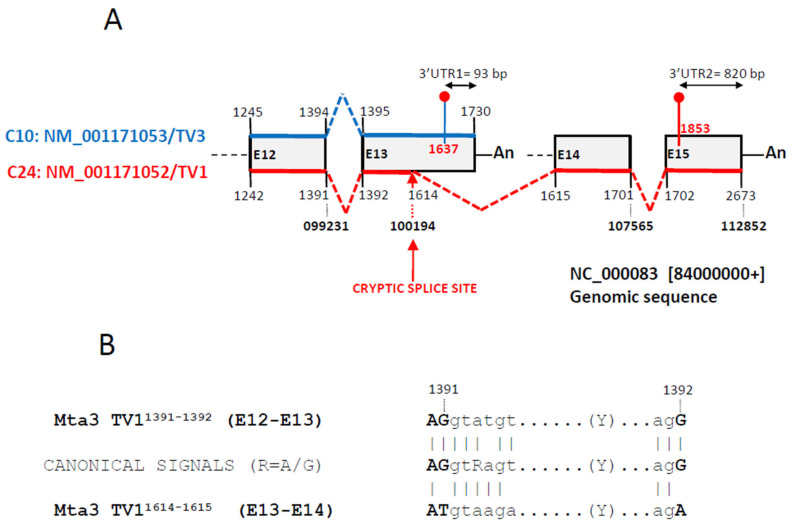
Alternative 5′ splicing event at the terminal region of the Mta3 transcript variant 1. (**A**) Diagram of four terminal exons (grey boxes) of the Mta3 gene. Shown are the last two exons of the transcript variant 3 (NM_001171053, blue at the top of the boxes) and the last four of transcript variant 1 (NM_001171052, red at the bottom of the boxes). Numbers state the beginning/end of each exon and dotted lines the splicing events linking exons. Bold numbers refer to the positions in the genomic sequence NC_000083, which includes the Mta3 gene. Shown are also the positions of the stop codons (red dots) and the poly-A tails (An). The cryptic splicing event links position 1614 in at the middle of exon 13 of variant 1 with position 1615 at exon 14 of variant 1. Exons E12–E15 are numbered (E12–E15) according to their positions in the genomic sequence NC_000083. Not drawn to scale. (**B**) Donor/acceptor splicing signals at exons E12/E13 (upper) and E13/E14 (alternative cryptic splicing, lower) of transcript variant 1 and comparison with the canonical signals. Coincident positions are signaled with a vertical bar. In bold, exonic sequences.

Lastly, we aimed the mechanisms causing length variations among the transcript variants described above. We first looked for the presence of alternative polyadenylation events [27], but none were detected. On the contrary, a careful analysis highlighted a major occurrence of alternative splicing events, including transcript variants generated by the activation of cryptic or latent splice donor/acceptor sites (Table 1 and Table 2). Figure 6A documents the complex splicing of the 3′UTR of Mta3 in which the activation of an alternative 5′ splice donor site at the middle of exon 13 and a far downstream acceptor at exon 14 led to the generation of a new transcript variant in C24. This new isoform had a longer 3′UTR of 820 bp vs. 93 bp in the transcript variant 3 expressed in C10. Furthermore, the length of the CDS was also affected by the activation of the new splice site, from 1542 bp long in variant 3 from C10 to 1757 bp long for variant 1 from C24. Figure 6B shows the alternative splice donor/acceptor signals activated in exons 13 and 14, which differ with the canonical donor/acceptor sites and the donor/acceptor signals at constitutive splicing among exons 12 and 13.

## 4. Discussion

The sequencing revolution has led to the discovery of new families of regulatory RNAs (lncRNAs, miRNAs, piRNAs, etc.) that establish complex regulatory networks with mRNAs. Alterations in these networks contribute to the disease process and could be also considered as targets of therapeutic intervention [28]. Among these new RNAs, miRNAs have an important role by regulating mRNA stability and function through the targeting of complementary sequences at the 3′UTR of mRNAs. Nevertheless, recent reports are showing that the relationship miRNA/mRNA is much more complex than previously described, since mRNAs avoid miRNA targeting by regulating the length and sequence of their 3′UTRs by alternative polyadenylation or through the regulated use of alternative 3′ terminal exons [3,27]. In this context, we aimed to study the variability in the 3′UTRs of DEGs during ATS progression, or regression after treatment with an αCD40-specific siRNA, in aortas from ApoE-deficient mice. Our original microarray expression data identified mRNAs up- or downregulated in our experimental conditions (see Figure 1 and [23]), and here we report the results of the analysis of the downregulated transcripts. In this work, we took advantage of the double identification of DEGs in the microarray output that included the “GeneSymbol”, a gene identifier common to all isoforms, and the “Seqname”, an individual isoform identifier.

**Figure 7:**
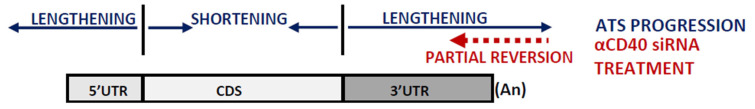
Graphical summary of the findings of this work. Graphical representation of the functional regions (5′UTR, CDS, 3′UTR and poly-A tail) of a model mRNA and their length dynamics during ATS progression and upon treatment with the αCD40 siRNA. Arrows and headings indicate the sense of the length variations. Designed from data in Figure 2, Figure 3 and Figure 4. Not drawn to scale.

Our results draw a complex picture of sequence length alterations in ATS progression since these were not restricted to 3′UTRs but also affected 5′UTRs and the Coding Regions of mRNAs (Figure 7). In this sense, we found a clear lengthening of 3′UTRs during disease progression (C10 to C24, Figure 2A) that was partially reversed by the siRNA treatment (C10 vs. T10 and C24 vs. T24, Figure 2). Variations in 3′UTR length would cause the acquisition of new regulatory potentials with regard to stability, translational or subcellular localization by controlling the accessibility of miRNAs or of RBPs to their target sequences [3,29] and have been mostly linked to Alternative Polyadenylation (APA) events regulated by general or type-specific factors [30]. In this way, 3′UTR lengthening would increase the regulatory potential by including miRNA or RBP-responsive elements that in tumor-suppressor genes have been associated with cancer resistance by increasing the density of mRNA-stability-regulating RBPs [31], while the shortening of 3′UTRs has been linked to tumor progression [32,33] by releasing oncogene transcripts from miRNA control.

On the other hand, we also describe a small but significant lengthening of 5′UTRs from week 10 to week 24 of ATS progression (Figure 3A) not reversed by the antiCD40 treatment (Figure 4A). 5′UTR length variations have been linked to the activation of alternative or upstream start codons [34], of alternative promoters [35] or alternative splicing events [36]. Furthermore, 5′UTRs have been shown to be targeted by miRNAs [37,38] in a way depending on their secondary structure [39].

We next addressed the mechanisms promoting sequence lengthening by studying transcript isoform switching in ATS progression or regression as a way to describe evolution of gene expression. We were able to detect nine different genes that were simultaneously expressed in two conditions (six genes in C10 and C24, another two in T10 and T24 and one common to both experiments), which, furthermore, showed isoform switching with a total of 19 transcript variants expressed in the different conditions (Table 1 and Table 2). In this group of transcript isoforms, we identified the alternative splicing of 5′ and 3′UTRs as a frequent mechanism contributing to the sequence variability. Furthermore, among these 19 transcripts, we found two, Mta3 and Ly6e, expressing alternative 3′ or 5′UTRs, respectively. In the case of Mta3, this involved the activation of a cryptic/latent donor splice site [40] at the middle of one exon (Figure 6). This is interesting because alternative splicing has been considered as a minor contributor to the generation of transcript variability since the analysis of the superfamily of odorant receptor (OR) genes showed that over 80% of OR mRNAs were submitted to alternative polyadenylation while only a few of these used alternative splicing to generate length variants of 3′UTRs [41].

The regulation of alternative splicing is very complex and involves multiple regulatory sites at the pre-mRNAs (splicing donor and acceptor sites, splicing enhancers and silencers, etc.) recognized by mRNA-binding proteins and U-snRNPs ([42] for review). The first step in the maturation of an mRNA is the recognition of the 5′splice site through the functional integration of cis-acting splice signals and splicing regulatory elements (SREs) in the mRNA with the activity of U1-snRNPs and a number of trans-acting splicing factors, all of them working in the context of the secondary structure of the mRNA, which regulates the accessibility of the splicing machinery to the splice site [43]. Nevertheless, in the genome, there are many potential exonic 5′ splicing sites that are not used under physiological conditions, raising the interesting question of the nature of the mechanisms restricting 5′SS selection in normal cells and how these mechanisms are altered in disease to allow the recognition of cryptic or latent 5′SS. This is especially important because the improper activation of latent splice sites could result in the incorporation of intronic sequences with potential premature in-frame stop codons to mature mRNAs [44]. In this sense, a recent model of 5′SS selection by Brillen and colleagues demonstrated that SREs and SRE-binding proteins were able to block “weak” cryptic 5′splice sites to facilitate the recognition of “strong” actual sites following a sequential, iterative and position-dependent process [45], while Boehm and colleagues proposed the Exon Junction Complexes to suppress cryptic 5′splice sites through the recruitment of the splicing regulator RNPS1 [46]. Furthermore, spliceosomes with noncanonical U1-snRNAs or changes in the stoichiometry of spliceosome components could also contribute to the recognition of variant 5′ss to generate cell/tissue-specific patterns of alternative splicing [47,48], and Arafat and Sperling have proposed a quality control mechanism (SoS, suppression of splicing) that would distinguish among normal and latent 5′ splice sites and suppress these last through the recognition of a functional ORF (see [49] for a recent review). Lastly, an interesting mechanism has been proposed by Movassat and colleagues that links the recognition of the 5′SS in the last exon with the cleavage and polyadenylation site and identifies PA factor CstF64 as a potential regulator of alternative splicing [50]. In this context, it is clear that changes in the expression of splicing factors during ATS progression could have an impact on the regulation of the alternative splicing events here described, and work is currently in progress in our group to probe this hypothesis. This work has exposed isoform switching and alternative splicing as contributors to the generation of transcript length variability in line with other authors who highlighted the involvement of mRNA isoform switching in physiological processes and diseases such as aging [51], cancer [52] or severe Covid [53], among others. Furthermore, here, we have also identified a set of transcripts that can be studied to validate their own roles and the roles of splicing-regulating factors and splicing alterations in ATS progression. Clearly, splicing is becoming a subject of intense research to develop therapies aimed to normalize splice-site usage, and anomalous alternative splicing events, and to correct variations in the expression of splicing factors [54]. In this sense, antisense oligonucleotides have been used to normalize aberrant splicing by targeting specific mRNA isoforms for degradation or translational repression or by inducing new stop codons [55] but also to target splice sites to prevent splicing factor binding or spliceosome assembly [56], and small molecules have also been used to inhibit the splicing machinery [57].

On the other hand, a number of points could hinder the relevance of this work, starting with the use of microarray hybridization instead of RNA-seq to obtain our expression data [23]. Microarrays represent a quite static view of gene expression, being limited by probe design and by the selection of the arrayed mRNA isoforms, and we could not discard the presence of additional changes in the patterns of mRNA-alternative polyadenylation/splicing in our mRNAs, not represented among the microarray probes. Clarifying this interesting point will require the use of RNA-seq data to identify all actual isoform variants. Next, we should also take into account the effect of possible confounders such as aging, although, in this regard, this study was designed to end when the mice were 24 weeks old, which is relatively young to suffer age-related alterations. Lastly, we cannot evaluate the relevance of these length variations with regard to ATS progression mainly because the research project was not designed to answer this question. Nevertheless, here, we report the identification of a number of individual transcripts that can be studied to determine the relevance of length variations and splicing alterations on the development of ATS.

In conclusion, here, we have characterized the length dynamics of transcript variants downregulated during ATS progression. We have uncovered general length variations, not only at the 3′UTRs of these transcripts as expected, but also at their 5′UTR and CDS, and have given evidence for the involvement of alternative splicing as a frequent mechanism for the generation of length variability. To the best of our knowledge, this is the first report linking ATS development with changes in the length of ATS-DEGs, and it highlights splicing factors as possible targets of therapeutical intervention in ATS progression.

## Figures and Tables

**Table 1:** Length variations in transcript variants differentially expressed in C24 vs. C10. Shown are the genes (Genesymbol) expressed in both C10 and C24, the transcript variants newly expressed in C24 (vs. C10), and the length variations of their full-length mRNA sequences (FL) and their functional regions 5′UTR, CDS and 3′UTR. [S] The isoform/region expressed in C24 was shorter than those at C10. [L] The isoform/region expressed in C24 was longer than those at C10. [=] No length differences among C10 and C24 were found. (1) Expression of these two variants was repressed in C24. (2) Use of an alternative 3′ acceptor results in single-codon skipping at an exon–exon junction at the 5′UTR of NM_11252 (vs. NM_001166623). Gene and transcript variant identities are as follows: Dusp22: dual-specificity phosphatase 22; Ly6e: lymphocyte antigen 6 family member E; Mta3: metastasis-associated 3; Rab11fip5: RAB11 family-interacting protein 5 (class I); Rbmx: RNA-binding motif protein, X chrom.; Skor1: SKI family transcriptional corepressor 1; Srsf5: serine and arginine-rich splicing factor 5. See Supplementary Table S1 for more details.

Genesymbol	Isoforms in C10 (Seqname)	New Isoforms in C24 (Seqname)	Length Variations (C24 vs. C10) in:				Mechanisms of Length Variation
			FL	5′UTR	CDS	3′UTR	
Dusp22	NM_001037955	NM_134068	S	=	S	S	Cryptic splicing at the end of the CDS-3′UTR generates a shorter NM_134068
Ly6e	NM_001164037 NM_001164038^1^ NM_001164040^1^	NM_001164037	L	L	=	=	Three alternative 3′Splice aceptor sites generate three different 5′UTRs
Mta3	NM_001171053	NM_001171052	L	=	L	L	An alternative 5′ splice donor site generates a new CDS and 3′UTR
Rab11fip5	NM_177466	NM_001003955	L	=	L	=	Inclusion of an alternative exon in the CDS of NM_001003955
Rbmx	NM_001166623	NM_011252	S^2^	S^2^	=	=	Shorter 5′UTR in NM_011252 by activation of an alternative 3′ splice acceptor site at an exon–exon junction at the 5′UTR
Skor	NM_001163757	NM_001163758	S	L	S	=	Shorter CDS in NM_001163758 than in NM_001163757 through use of two alternative start codons
Srsf5	NM_001079694	NM_009159	S	S	=	=	4 nts missing in the 5′UTR of NM_009159

**Table 2:** Length variations in transcript variants differentially expressed in T24 vs. T10. Shown are the genes (Genesymbol) expressed in both T10 and T24, the transcript variants newly expressed in T24 (vs. T10) and the length variations of their full-length mRNA sequences (FL) and their functional regions 5′UTR, CDS and 3′UTR. [S] The isoform/region expressed in C24 was shorter than those at C10. [L] The isoform/region expressed in C24 was longer than those at C10. [=] No length differences among C10 and C24 were found. (1) Use of an alternative 3′acceptor results in single-codon skipping at an exon–exon junction at the 5′UTR of NM_11252 (vs. NM_001166623). Gene and transcript variant identities are as follows: Ilf3: interleukin enhancer-binding factor 3; Pwwp2a: PWWP-domain-containing 2a; Rbmx: RNA-binding motif protein, X chrom. See Supplementary Table S2 for more details.

Genesymbol	Isoforms in T10 (Seqname)	New Isoforms in T24 (Seqname)	Length Variations (T24 vs. T10) in:				Mechanisms of Length Variation
			FL	5′UTR	CDS	3′UTR	
Ilf3	NM_001042708	NM_001042707	S	=	L	S	Alternative exons in CDS and 3′UTR. Alternative splicing (METEs)
Pwwp2a	NM_027557	NM_001164231	L	=	S	L	An alternative 5′ splice donor site generates a new CDS and 3′UTR
Rbmx	NM_001166623	NM_011252	S^1^	S^1^	=	=	Shorter 5′UTR in NM_011252 by activation of an alternative 3′ splice acceptor site at an exon–exon junction at the 5′UTR

## Data Availability

The original contributions presented in the study are included in the article, further inquiries can be directed to the corresponding authors.

## Supplementary Materials

The following supporting information can be downloaded at: https://www.mdpi.com/article/10.3390/biomedicines12122703/s1, Table S1: Length variations in transcript isoforms expressed from C10 to C24. Shown are the genes (Genesymbol) common to the two experimental conditions, their expressed transcript variants, the lengths of the 5’UTR, CDS, 3’UTR and mRNA of these transcript variants. Also shown are their expression levels (as the absolute value of the Fold Change, FC, and the statistical significance. Last column highlights the mechanism regulating length variation. In bold, newly expressed transcript variants; Table S2: Length variations in transcript isoforms expressed from T10 to T24. Shown are the genes (Genesymbol) common to the two experimental conditions, their expressed transcript variants, the lengths of the 5’UTR, CDS, 3’UTR and mRNA of these transcript variants. Also shown are their expression levels (as the absolute value of the Fold Change, FC, and the statistical significance. Last column highlights the mechanism regulating length variation. In bold, newly expressed transcript variants.

## Abbreviations

5′SS	5′ splice site
3′SS	3′ splice site
APA	Alternative polyadenylation
ATS	Atherosclerosis
C10/24	Mice treated with the scrambled control siRNA at weeks 10/24
CDS	Coding sequence
ceRNA	Competing endogenous RNA
CPSF	Cleavage and polyadenylation specificity factor
CstF64	Cleavage stimulation factor 64 kDa
DEG	Differentially expressed gene
PA	Polyadenylation
PABPN	Poly(A)-Binding Protein Nuclear 1
piRNA OR	Piwi-associated RNA Odorant receptor
RBP	RNA-binding protein
RNPS1	RNA-Binding Protein with Serine-Rich Domain 1
SRE	Splicing regulatory element
SoS	Suppressor of splicing
U1-RNPs	U1-ribonucleoprotein
T10/24	Mice treated with αCD40 siRNA at weeks 10/24
