# Supplementary material for: Generation of Transcript Length Variants and Reprogramming of mRNA Splicing During Atherosclerosis Progression in ApoE-Deficient Mice
Source: Biomedicines. 2024 Nov 26;12(12):2703. doi: 10.3390/biomedicines12122703 (PMC11672872; doi:10.3390/biomedicines12122703)
Supplement: Supplementary file 1 [file biomedicines-12-02703-s001.zip › biomedicines-3243757-supplementary.pdf]

Genesymbol	Transcript isoforms in		Length (in bp) of				Expression	
	C10 (Seqnames)	C24 (Seqnames)	mRNA	5'UTR	CDS	3'UTR	FC(abs)	P
**Dusp22**	[1]NM_001037955		3058	67	615	2376	2.20	0.021
		[1]NM_001037955					3.03	0.006
		**[2]NM_134068**	1070	42	552	476	3.08	0.016
**Ly6e**	[3]NM_001164038		2095	511	390	1194	2.57	0.035
	[4]NM_001164037		2179	595	390	1194	3.51	0.021
	[5]NM_001164040		1944	360	390	1194	3.90	0.020
		[4]NM_001164037					2.75	0.041
**Mta3**	[6]NM_001171053		1740	95	1542	93	2.03	0.035
		**[7]NM_001171052**	2673	95	1758	820	7.10	1.0e-05
**Rab11fip5**	[8]NM_177466		4137	106	1935	1904	2.94	0.007
		[8]NM_177466					2.34	0.016
		**[9]NM_001003955**	6156	106	3954	2096	2.02	0.019
**Rbmx**	[10]NM_001166623		2057	156	1173	728	9.08	0.014
		[10]NM_001166623					8.88	0.015
		**[11]NM_011252**	2054	153	1173	728	4.14	0.037
**Skor**	[12]NM_001163757		3613	98	2805	710	2.04	0.019
		**[13]NM_001163758**	3597	112	2775	710	2.74	0.0009
**Srsf5**	[14]NM_001079694		1493	158	807	528	2.04	0.019
		[14]NM_001079694					2.96	0.001
		**[15]NM_009159**	1489	154	807	528	2.52	0.001

Supplementary Table S1. Length variations in transcript isoforms expressed from C10 to C24. Shown are the genes (Genesymbol) common to the two experimental conditions, their expressed transcript variants, the lengths of the 5'UTR, CDS, 3'UTR and mRNA of these transcript variants. Also shown are their expression levels (as the absolute value of the Fold Change, FC, and the statistical significance. Last column highlights the mechanism regulating length variation. In bold, newly expressed transcript variants.

Gene and transcript variant identities are as follows:

[Dusp22]: Dual specificity phosphatase 22: (1) variant 1 mRNA, (2) variant 2 mRNA; [Ly6e]: Lymphocyte antigen 6 family member E: (3) variant 4 mRNA, (4) variant 3 mRNA, (5) variant 6 mRNA; [Mta3]: Metastasis associated 3: (6) variant 3 mRNA, (7) variant 1 mRNA; [Rab11fip5]:

RAB11 family interacting protein 5 (class I): (8) variant 2 mRNA, (9) variant 1 mRNA; [Rbmx]: RNA binding motif protein, X chrom.: (10) variant 3 mRNA, (11) variant 1 mRNA; [Skor1]: SKI family transcriptional corepressor 1: (12) variant 3 mRNA, (13) variant 4 mRNA; [Srsf5]: Serine and arginine-rich splicing factor 5: (14) variant 2 mRNA, (15) variant 3 mRNA.

SUPPLEMENTARY TABLE S1

Genesymbol	Transcript isoforms in		Length (in bp) of				Expression	
	T10 (Seqnames)	T24 (Seqnames)	mRNA	5'UTR	CDS	3'UTR	FC(abs)	P
**Ilf3**	[1]NM_001042708		3777	324	2148	1305	3.46	0.039
		[1]NM_001042708					3.92	0.019
		**[2]NM_001042707**	3564	324	2694	546	5.67	0.001
**Pwwp2a**	[3]NM_027557		3251	66	2190	995	3.44	0.011
		[3]NM_027557					11.55	0.002
		**[4]NM_001164231**	3855	66	1599	2190	2.90	0.043
**Rbmx**	[5]NM_001166623		2057	156	1173	728	8.89	0.016
		[5]NM_001166623					10.06	0.013
		**[6]NM_011252**	2054	153	1173	728	4.18	0.037

Supplementary Table S2. Length variations in transcript isoforms expressed from T10 to T24. Shown are the genes (Genesymbol) common to the two experimental conditions, their expressed transcript variants, the lengths of the 5'UTR, CDS, 3'UTR and mRNA of these transcript variants. Also shown are their expression levels (as the absolute value of the Fold Change, FC, and the statistical significance. Last column highlights the mechanism regulating length variation. In bold, newly expressed transcript variants.

Gene and transcript variant identities are as follows:

[Ilf3]: Interleukin enhancer binding factor 3: (1) variant 3 mRNA, (2) variant 2 mRNA; [Pwwp2a]: PWWP domain containing 2a: (3) variant 2 mRNA, (4) variant 1 mRNA; [Rbmx]: RNA binding motif protein, X chrom.: (5) variant 3 mRNA, (6) variant 1 mRNA.
